# Antimicrobial nano-silver non-woven polyethylene terephthalate fabric via an atmospheric pressure plasma deposition process

**DOI:** 10.1038/srep10138

**Published:** 2015-05-07

**Authors:** Xiaolong Deng, Anton Yu Nikiforov, Tom Coenye, Pieter Cools, Gaelle Aziz, Rino Morent, Nathalie De Geyter, Christophe Leys

**Affiliations:** 1 Department of Applied Physics, Ghent University, Sint-Pietersnieuwstraat 41B4, 9000 Gent, Belgium; 2 Department of Pharmaceutical Analysis, Ghent University, 9000 Gent, Belgium

## Abstract

An antimicrobial nano-silver non-woven polyethylene terephthalate (PET) fabric has been prepared in a three step process. The fabrics were first pretreated by depositing a layer of organosilicon thin film using an atmospheric pressure plasma system, then silver nano-particles (AgNPs) were incorporated into the fabrics by a dipping-dry process, and finally the nano-particles were covered by a second organosilicon layer of 10-50 nm, which acts as a barrier layer. Different surface characterization techniques like SEM and XPS have been implemented to study the morphology and the chemical composition of the nano-silver fabrics. Based on these techniques, a uniform immobilization of AgNPs in the PET matrix has been observed. The antimicrobial activity of the treated fabrics has also been tested using *P. aeruginosa*, *S. aureus* and *C. albicans*. It reveals that the thickness of the barrier layer has a strong effect on the bacterial reduction of the fabrics. The durability and stability of the AgNPs on the fabrics has also been investigated in a washing process. By doing so, it is confirmed that the barrier layer can effectively prevent the release of AgNPs and that the thickness of the barrier layer is an important parameter to control the silver ions release.

Non-woven polyethylene terephthalate (PET) has been used in a wide range of applications due to its outstanding characteristics such as excellent mechanical strength and good chemical stability.^1^ In recent years, a lot of attention has however been paid to achieve a more multifunctional performance of PET fabrics, especially in the health and hygienic field. For this purpose, PET fabric treated with antimicrobial agents has been extensively studied and results indicate the capability of preventing the growth of pathogenic microorganisms, such as bacteria, fungi, algae,….^2^,^3^,^4^ Various antimicrobial agents (silver, copper, metal salts, quaternary ammonium compounds, polyhexamethylene biguanides, triclosan biopolymer chitosan, N-halamine,…) have already been used as an antimicrobial finish on textile materials.^5^,^6^,^7^,^8^,^9^,^10^,^11^,^12^

Among the above mentioned antibacterial chemicals, silver has been widely used due to its broad spectrum of antibacterial activity and its low toxicity towards mammalian cells.^13^ The release of silver ions is believed to be the main reason for its antibacterial properties. Ionized silver is highly active, as it binds to tissue proteins and brings structural changes to the bacterial cell wall and nuclear membrane leading to cell distortion and death.^14^ Due to their small size, silver nanoparticles (AgNPs) have emerged as a new generation of antibacterial agents with unique properties for diverse medical applications.^15^ Unfortunately, the direct interaction of AgNPs with human cells inevitably leads to cytotoxicity and genotoxicity.^16^ Taking into consideration the above mentioned concern, it is of crucial importance to immobilize AgNPs onto the substrate material. In this way, release of AgNPs in real-life applications can be completely avoided and only a controlled release of silver ions can be achieved. This immobilization approach has been intensively studied since it can suppress the potential hazardous influence of the silver nanoparticles while still having the beneficial antibacterial properties.^17^

In the past, different strategies have already been proposed and investigated for the incorporation of silver nanoparticles on a fabric. Deposition of a metallic silver film on PET fabrics has been studied using magnetron sputtering and an ion-beam-assisted deposition process.^18^,^19^,^20^ Methods of dipping-pad-drying for the silver finishing of non-woven fabrics using colloidal silver have also been applied.^21^,^22^ Some of the above mentioned methods are however based on reactions in liquid medium and require surfactants, reducing agents or templates for the synthesis of the silver nanoparticles, resulting in the inevitable presence of impurities in the final products.^23^ Moreover, due to the poor adhesion between the organic fabrics and the inorganic particles caused by their difference in surface energy, surface modification of fabrics or nanoparticles is important or even essential when incorporating nanoparticles. 24 In addition, weakly bonded AgNPs will affect antibacterial efficiency and even cause potential cytotoxicity. The method of film deposition on non-woven fabrics with immobilized silver nanoparticles is of great interest but has not been tested before. Plasma polymerization is a very promising deposition technique because of the environmental safeness, sustainability and high deposition rate coupled with good film adhesion to different substrates. Moreover, materials with various structures, flat or complex 3D objects, can be used as a substrate.

In this work, PET non-woven fabrics with antimicrobial properties will be produced by firmly immobilizing silver nanoparticles via a double layer of a plasma deposited organic film. This process was carried out by a three-step procedure as shown in Fig. 1. At first, an organosilicon thin film was deposited on the surface of the fabrics using a plasma jet deposition system. This first layer, called through the paper “the reservation layer”, is used for the silver immobilization and to control the silver nanoparticles adhesion to the PET fibres. Then the fabric was immersed into an AgNPs suspension for the incorporation of silver, and the color of the fabric changed from white to gray (Fig. 2). In the final step of the process, a second layer of organosilicon film was deposited using the plasma jet system.

To the best of our knowledge, this is the first time that immobilization of silver nanoparticles on non-woven fabrics is studied by using a double organosilicon layer obtained with an atmospheric pressure plasma process. In order to have a better understanding of the capability of the proposed process, the chemical composition and morphology of the silver decorated fabrics will be characterized by X-ray photoelectron spectroscopy (XPS) and scanning electron microscopy (SEM) respectively. The antimicrobial activity of the samples will also be tested against three common pathogenic microorganisms, *Pseudomonas aeruginosa* (*P. aeruginosa*), *Staphylococcus aureus* (*S. aureus*) and *Candida albicans* (*C. albicans*). The antimicrobial efficiency of the treated fabrics in static experiments and under mechanical load represented by washing will be analyzed and discussed in detail.

## Results and discussions

### Plasma polymerization on non-woven PET fabrics

Plasma polymerization is capable of producing ultra-thin, polymer-like layers with a defined, regular structure on the top surface of flat materials. However, in this work, non-woven porous materials are exposed to a plasma polymerization process. Therefore, it is very important to study the penetration efficiency of the plasma polymerization process through the PET non-woven after the deposition of the first organosilicon layer. For this purpose, plasma polymerized PET samples are examined with XPS on two sides: the top side which is directly exposed to the plasma and the bottom side, which is in close contact with the sample holder.

The atomic composition of the untreated and the plasma treated PET non-woven fabric is determined using XPS and is shown in Table 1. The raw fabric is composed of 73.4% carbon and 26.6% oxygen. For the samples after plasma polymerization, the surfaces on the top and bottom sides show the same components: carbon, oxygen and silicon. It is noteworthy that the concentrations of the elements on both sides are almost identical. Thus, the plasma clearly penetrates into the structure of the fabric and plasma deposition can be achieved in the non-woven bulk as well as on the bottom side of the fabric. This can be explained by the efficient transfer of active plasma species by the vertical gas flow along the plasma jet.

**Figure 3:**
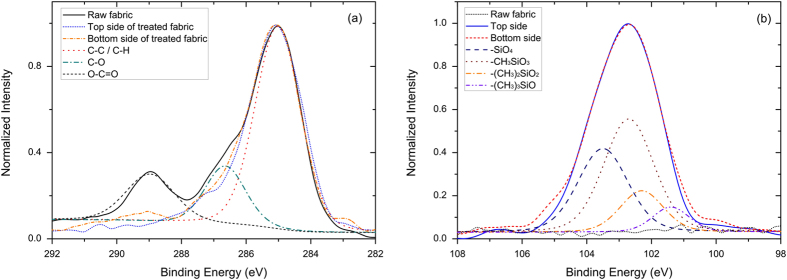
XPS high resolution analyses of plasma-polymerized fabrics. (**a**) High resolution C1s spectra for the raw PET fabric with the top and bottom side surface of plasma-polymerized fabrics. The C1s peak of raw PET fabric has been deconvoluted considering 3 groups; (**b**) High resolution Si2p spectra for the raw PET fabric with the top and bottom side surface of plasma-polymerized fabrics. The Si2p peak for plasma treated surface has been deconvoluted considering 4 groups.

Figure 3 shows the C1s spectra and Si2p spectra of the raw fabric and the top/bottom side of the plasma treated PET non-woven. These high resolution XPS spectra can be utilized to analyze the chemical state of the elements present on the surfaces. As shown in Fig. 3 (a), the profiles of the C1s peak reveal differences between the raw fabric on the one hand and the treated fabrics on the other hand which suggests the presence of completely different bonds on the surface after plasma polymerization. The C1s spectrum of the original PET fabric can be decomposed into three components: a component at 285.0 ± 0.1 eV due to C-C or C-H bonds, a component at 286.5 ± 0.1 eV due to C-O bonds and a component at 289.1 ± 0.1 eV due to O-C=O bonds. In contrast, the C1s spectra of the plasma treated samples have a completely different profile: these surfaces no longer contain O-C=O bonds at 289.1 eV but do contain a small peak at 283.1 eV suggesting the presence of Si-C bonds. These C1s spectra thus indicate that the fabric surface has been covered with an organosilicon layer due to the plasma polymerization process. As shown in Fig. 3 (b), the raw fabric does not contain any Si2p peak. However, this peak is present for the plasma polymerized samples suggesting again the successful deposition of an organosilicon film on the PET fabric. The Si2p spectra shown in Fig. 3 (b) can be deconvoluted into 4 components: a component at 105.eV ± 0.1 eV due to (CH_3_)_3_SiO, a component at 102.2 ± 0.1 eV due to (CH_3_)_2_SiO_2_, a component at 102.8 ± 0.1 eV due to CH_3_SiO_3_ and a component at 103.4 ± 0.1 eV due to SiO_4_.^25^ Starting from pure TMDSO, one would expect to only observe a peak at 102.2 eV but due to the addition of a small amount of oxygen in the discharge gas, a mixture of different silicon bonds is present in the plasma deposited films. It is also important to note that the C1s and Si2p spectra of the top and bottom side of the plasma treated fabric have almost identical profiles confirming uniform chemical composition of the deposits throughout the PET fabric.

### Chemistry and morphology of the treated materials at different process steps

The chemical composition of the samples in different experimental steps was analyzed with XPS and results are presented in Table 2. In the dipping and drying step, silver has been incorporated into the fabrics with a silver concentration of 1.9% on the surface. Oxygen and silicon concentrations decrease after incorporation of the AgNPs which is in good agreement with our previously published research^25^. Silver concentration on the surface is reduced to 1.1% and 0.5% after deposition of a 10 nm and 50 nm barrier layer respectively. In our opinion, this effect is due to the blocking of the silver signal by the barrier layer surface. It is well know that XPS can be used to study the chemistry of the top 10 nm of a surface. As a result, one would expect to detect no silver concentration when a 10 nm or a 50 nm barrier layer is deposited on top of the AgNPs. However, in Table 2, a small silver concentration is still present on the samples with the barrier layers on top. This effect can be attributed to the presence of cracks, pores or thin covering places on the points of AgNPs incorporation. While the reasons for the formation of the cracks and pores are not yet completely clear, it could be the result of thermal stress induced during the plasma deposition since a strong temperature gradient exists between the gas phase and the cold substrate. The presence of those imperfections in the barrier layer can be indirectly observed by the change of the Si signal in the XPS measurements. In the dipping and drying step, the silicon concentration is reduced from 21.6% to 15.2% due to the incorporation of the AgNPs. With the deposition of a 10 nm barrier layer, the Si concentration recovered to 19.9%. This value is lower than the value for the initial organosilicon film (21.6%) which suggests that the surface of the AgNPs was not completely covered by the 10 nm organosilicon film and that some imperfection areas are present in the thin barrier layer. Increasing the thickness of the barrier layer to 50 nm causes a further increase of the silicon percentage to 21.1%, which is close to the initial value due to the reduction of those imperfections. Thus, the imperfections in the 10 nm barrier layer can be clearly detected by XPS and can be considerably reduced by increasing the barrier thickness to 50 nm. Correspondingly, the thickness of the barrier layer is expected to be a key parameter in the control of the coating performance and the release of silver through imperfections.

**Figure 4:**
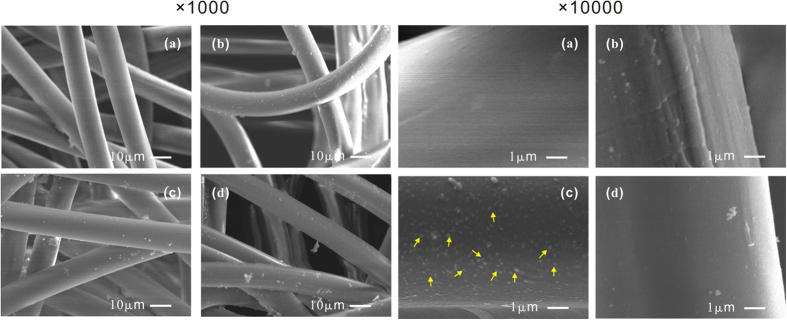
SEM image of the samples at different preparation steps. Two sets of SEM images with magnification of × 1000 (acceleration voltage:15 kV) and × 10000 (acceleration voltage 10 kV). (**a**) SEM images of original PET fabric; (**b**) the fabrics with a reservation layer, (**c**) the fabric after incorporation of AgNPs (labeled by some arrows on the image with × 10000 magnification), (**d**) the fabric with a 50 nm barrier layer.

Analysis of AgNPs incorporation on PET fibers was carried out with SEM. Figure 4 represents the surface morphology of the samples at different fabrication steps: (a) raw PET fabric, (b) deposition of a reservation layer; (c) incorporation of AgNPs; (d) deposition of a barrier layer (50 nm). SEM figures with two different magnifications, i.e. × 1000 and × 10000, are presented on the left and right side respectively. The raw PET fabric consists of fibers with an average diameter of approximately 10 *μ*m having a smooth surface as shown in Fig. 4 (a). After being treated with the first plasma deposition process (Fig. 4 (b)), the surface of the PET fibers is completely covered by a film. Figure 4 (c) is made after the dipping and drying step. Magnification of the surface reveals successful incorporation of silver particles on the fibers with a diameter of approximately a few tens of nanometers. A dramatic change of the surface morphology is observed after the barrier layer deposition. The surface of the PET fibers shown in Fig. 4 (d) is more smooth, which indicates the covering of the incorporated particles by a new film layer.

### Antimicrobial efficiency of the treated PET materials

To test the antimicrobial efficiency of the PET fabrics, the following test organisms were used: *Pseudomonas* aeruginosa (*P. aeruginosa*) ATCC 9027, *Staphylococcus aureus* (*S. aureus*) Mu50 and *Candida albicans* (*C. albicans*) SC5314, which are corresponding to a gram-positive bacteria, a gram-negative bacteria and a fungus respectively. *P. aeruginosa* infections are a serious problem in patients hospitalized with cancer, cystic fibrosis and burns; with high fatality rates.^26^ *P. aeruginosa* is naturally resistant to a wide range of antibiotics. *S. aureus* is a pathogenic microorganism causing many diseases such as toxic shock syndrome, superficial skin lesions, deep-seated infections and is the leading overall cause of hospital acquired (nosocomial) infection of surgical wounds.^26^ Moreover, it is resistant to a great number of antimicrobial agents.^27^ *C. albicans* is the most common cause of opportunistic fungal infections and it can cause infections that range from superficial infections of the skin to life-threatening systemic infections.^28^ Since all of the three microorganisms are considered as common potential pathogens for infections, those three aforementioned microorganisms were selected for evaluation of the antimicrobial efficiency in this research.

Percent reduction of organisms R which indicates biostatic efficiency resulting from contact with the sample was determined using the following formula:
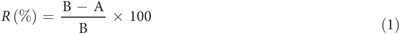
where A is CFU per milliliter for the medium with the treated substrate after incubation, and B is CFU per milliliter of the medium with the control samples after incubation. The control samples in this part are corresponding to samples with the deposition of the first film layer.

**Figure 5:**
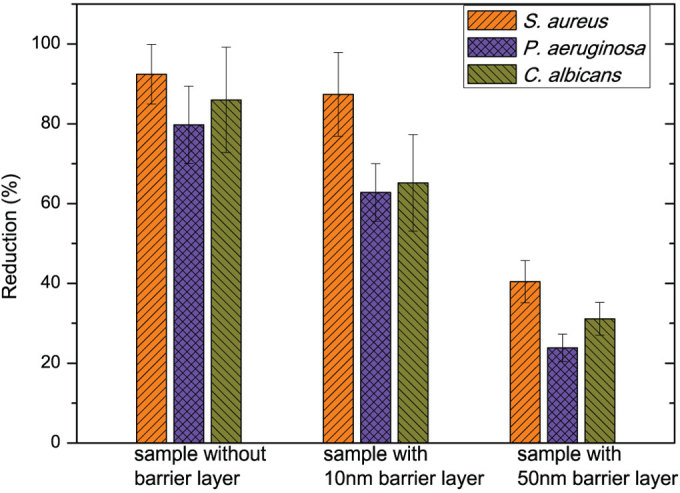
Antimicrobial activity of the samples against three microorganisms. The reductions of the bacteria with the control sample, the treated sample with a 10 nm barrier layer and the sample with a 50 nm barrier layer.

The antimicrobial properties of the samples were tested against *P. aeruginosa*, *S. aureus* and *C. albicans*. Initial sample with a 70 nm organosilicon film having no antimicrobial activity was used as a control. Antimicrobial capability of the treated PET fabrics without a barrier layer and with a 10 nm and 50 nm barrier layer is shown in Fig. 5. All samples with AgNPs exhibit antimicrobial activity against the three microorganisms, which clearly indicated that the growth of microorganisms in medium was affected by the presence of AgNPs. All treated PET fabrics show higher efficiency against *S. aureus* and lower against *P. aeruginosa*, which is in agreement with the results on commercially available silver-containing dresses.^29^ The samples with AgNPs but without a barrier layer have shown highest reduction of more than 90% of *S. aureus* and *C. albicans* and 80% of *P. aeruginosa.* Presence of the barrier layer results in a decrease of the antimicrobial efficiency to almost 50% reduction in the case of a 50 nm barrier layer. Such a strong effect of barrier layer can be linked to the way how AgNPs induce the antimicrobial effect.

**Figure 6:**
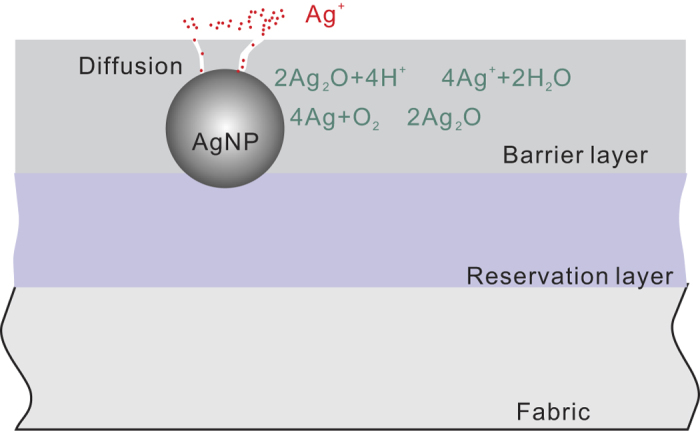
The scheme of silver ions generation and diffusion into a liquid medium. On the scheme the first layer, the reservation layer, is used for AgNPs immobilization and to control the silver nanoparticles adhesion to the PET fibres. The second layer, the barrier layer, is used for precise control over the release of silver ions. The generation of silver ions is proposed as 2 steps processes through silver oxidation.

At present, the exact mechanism of antimicrobial activity of silver is not clear. The release of silver ions is believed to be the main contribution to the effect. Ionic silver has a strong affinity to electron donor groups in biological molecules containing sulphur, oxygen or nitrogen. It is therefore able to bind to thiol (-SH) groups in enzymes, and inactivates them and destroys cell membranes as well.^30^ The DNA replication could also be inactivated due to the interaction with silver ions, as suggested by Thiel and Jung.^31^,^32^ It is well-known that silver nanoparticles can be oxidized after contact with the surrounding aqueous medium, either at the material-liquid interface or after water uptake by the polymer matrix. This results in the generation of silver ions, which diffuse to the liquid medium through the barrier layer, as shown in Fig. 6.

For the sample without the barrier layer, AgNPs on the materials have sufficient contact with the medium. Therefore, they can provide a fast release of silver ions into the medium and exhibit the strongest antimicrobial activity against microorganisms. When a barrier film is deposited, the direct contact between AgNPs and the medium is hampered and the release of silver ions from AgNPs is reduced. In this case, the Ag^+^ release is only possible through small cracks and pores in the barrier layer as it was shown in previous research.^33^ Moreover, the thickness of the barrier layer significantly influences the antimicrobial efficiency which is in contrast with work of L. Polux *et al.*^34^ In their study, a heptylamine (HA) matrix was used for the loading of AgNPs and it showed that the thickness has no significant effect on the release of Ag ions due to the nanoporous morphology structure of HA, which enables Ag ions to diffuse through it freely. In the plasma deposition experiments performed in our work, increasing the thickness of barrier layers leads to a reduction in the number of cracks and pores in the organosilicon matrix which can be a way to control antimicrobial efficiency and to obtain prolonged antibacterial effect in the end-user product.

Additionally to the control of the antibacterial efficiency through deposition of a barrier coating, it is also of crucial importance for applications to avoid any release of AgNPs during the life time of the textiles and/or under mechanical stress which occurs during wearing and washing of articles. The efficiency of the produced PET samples under mechanical stress will therefore be examined in detail in the following section.

### Antimicrobial efficiency under mechanical stress

**Figure 7:**
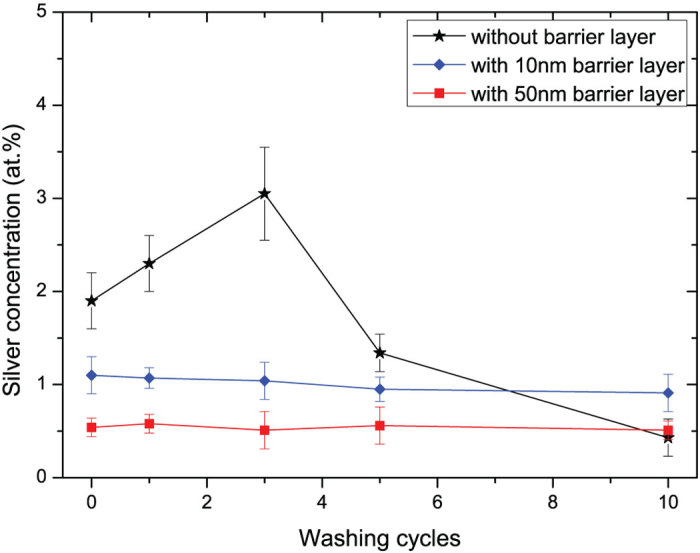
Silver concentration of the samples after mechanical washing cycles. The reservation layer thickness and amount of incorporated AgNPs is fixed through all the tests. The variation of the barrier layer thickness (0, 10, 50 nm) is used to prevent release of AgNPs in a series of washing cycles (1, 3, 5 and 10 washing cycles). Silver content is measured by XPS technique.

As mentioned, direct contact of AgNPs with cells should be completely avoided due to their potential toxicity. Cytotoxic effect of silver nanoparticles is mediated by the generation of oxidative stress resulting in a wide variety of physiologic and cellular events including stress, inflammation, DNA damage and apoptosis. In order to have those toxic effects, silver nanoparticles have to penetrate membrane and react with organelles, such as mitochondria. In our approach, the fixing AgNPs inside the coatings prevent the nanoparticles-cells reactions and further intracellular impact can be avoided. Therefore, we expect that the cytotoxicity of silver nanoparticles in the nanocomposite film is reduced. In this part, the durability of incorporated AgNPs in the materials is evaluated through a washing test of the samples after 1, 3, 5 and 10 washing cycles for 40 minutes each in a 200 ml of deionized water. All the samples were dried after washing in a vacuum prior to analysis. Figure 7 shows silver concentration on the top surface of three different samples after several washing cycles. The concentration shown in Fig. 7 is the average of 9 different locations on each sample. For the samples without a barrier layer, the silver concentration dramatically fluctuated with washing cycle. The interesting fact is that the concentration shows a steady rise to more than 3% for the first 3 washing cycles. Further washing processes lead to the reduction of silver concentration to about 1.3% and 0.4% after 5 and 10 washing cycles respectively.

The effect at the beginning of washing can be explained by loosening of fibers in the non-woven structure and fast desorption of physically absorbed particles with migration from bulk of the material to the surface. Correspondingly, the increase of Ag signal in XPS analysis for the material without barrier layer is attributed to the release of free AgNPs. The reduction of silver concentration in the following washing cycles is due to the loss of nanoparticles absorption on fibers surface and spreading in the water. In samples without barrier layer, silver thus exhibits non-uniform kinetics of release at the first 5 washing cycles. This confirms the dramatic desorption and separation of AgNPs from this fabric. For the samples with 10 nm and 50 nm barrier layers, silver concentration on the surface has almost negligible fluctuations for all washing cycles. This conclusion clearly proves our conceptions to the use of barrier layers to prevent any release of AgNPs from the matrix.

**Figure 8:**
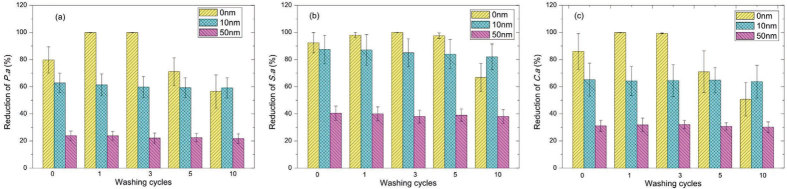
Antimicrobial activity of samples after mechanical washing cycles. The samples presented in fig. 7 are used: without a barrier layer, with a 10 nm barrier layer and a 50 nm barrier layer. After a series of washing cycles (1, 3, 5 and 10 washing cycles), the samples were tested for antimicrobial activity against: (a) *P. aeruginosa*; (b) *S. aureus*; (c) *C. albicans.*

The antimicrobial activity of treated fabrics with AgNPs in the absence and presence of a barrier layer was evaluated against *P. aeruginosa*, *S. aureus* and *C. albicans* after a series of washing cycles. The test results presented in Fig. 8 show that the general tendency of antimicrobial reduction is in perfect agreement with the XPS measurements of silver concentrations. For the samples without a barrier layer (orange bars in Fig. 8), a 100% reduction for the three microorganisms has been achieved after the first washing cycle. According to the XPS results in Fig. 8, this is due to a higher amount of AgNPs on the surface of the samples. This loose physical absorption of AgNPs can even cause the immigration of AgNPs into the bacterial suspension and result in a very high antimicrobial effect. This effect should however always be eliminated in any application. Moreover, after 5 times of washing, the antimicrobial activity reduced significantly due to the dramatic loss of AgNPs. For the samples with barrier layers (10 nm as blue bars and 50 nm as pink bars), the antimicrobial activity is maintained on a constant level and agrees with the XPS results, which confirms the stability of AgNPs bonding to the fibers and the positive effect of a barrier layer.

It is expected that samples with comparable silver concentrations would have similar antimicrobial activity. However, this is not always the case as observed for the samples after washing tests. According to the XPS analysis (Fig. 7), there were 0.4% and 0.5% of Ag on the samples without a barrier layer and the samples with a 50 nm barrier layer after 10 washing cycles respectively. However, as shown in Fig. 8, the bacterial reduction against three microorganisms of the sample without a barrier layer (orange bars) is higher than that of the sample with a 50 nm barrier layer (pink bars). In our opinion this phenomenon can be explained by the effect of the barrier layer preventing oxidation of AgNPs in liquid medium and release of silver ions. For the sample without a barrier layer after 10 washing cycles, all AgNPs on the surface were in direct contact with the medium and were affected by oxidation during the washing process which led to a high release of silver ions. While for the sample with a 50 nm barrier layer after 10 washing cycles, the release channels were limited to the cracks and pores on the barrier layer. Thus, even with comparable silver concentration on the surface for those two samples, the bacterial reduction was lower for the sample with a 50 nm barrier layer. Summarizing, barrier layer thickness has to be less than 50 nm if a high level of antimicrobial efficiency is required for the samples.

In summary, antimicrobial nano-silver PET fabrics were prepared through a three step process based on an atmospheric pressure plasma deposition process. It was revealed that the plasma can penetrate through the non-woven fabric structure and that a uniform double side deposition can be achieved. AgNPs were imbedded between two layers of organosilicon films: a reservation layer (1^st^ layer) and a barrier layer (2^nd^ layer). Variation of the barrier layer thickness is proposed as a novel precise method to control release of the silver ions and antimicrobial activity of the substrate. SEM and XPS results show that AgNPs can be uniformly distributed in the PET materials. The antimicrobial tests against *P. aeruginosa*, *S. aureus* and *C. albicans* revealed that the samples with a 10 nm barrier layer have stronger activity than those with a 50 nm barrier layer. The durability of silver nanoparticles bonding in the matrix as an important factor was also investigated through a washing process. The silver concentration in samples without a barrier layer showed a significant fluctuation after several washing cycles. This is explained by desorption of physically absorbed AgNPs from the non-woven materials and migration to the medium during the washing process. For the samples with a barrier layer, however, effective immobilization of silver in the matrix was confirmed with stability of the antibacterial effect even after 10 washing cycles. Thus, this work suggests that the double layer immobilization of AgNPs in non-woven fabrics using an organosilicon matrix can provide a precise way to control the release of silver ions with a high and lasting antimicrobial activity.

## Methods

### Plasma jet deposition system

The plasma jet consists of a pin and a mesh electrode placed in a quartz tube as described elsewhere.^35^ Investigations based on optical emission spectroscopy revealed that the nitrogen plasma contains abundant excited states of nitrogen molecules (N_2_ (A^3^Σ, B^3^Π, C^3^Π)) and ionized nitrogen species (N_2_^+^(B^2^Σ_u_, X^2^Σ_g_)). A plasma jet generated in N_2_ (7000 sccm) with admixing of O_2_ (20 sccm) and tetramethyldisiloxane (TMDSO, Sigma Aldrich) as the organosilicon precursor has been used for the deposition.^33^,^36^ The PET substrate is placed 10 mm away from the nozzle, where the gas temperature decreases to 310 K which is of practical importance for PET non-woven fabrics. The plasma head is mounted on a robotic arm (Stepcraft 300, Germany) in order to achieve a large scale treatment. The uniformity of the scanning process for large area deposition has been confirmed using silicon wafers as substrate.

### Preparation of silver loaded PET fabrics

Nano-silver non-woven PET fabrics were prepared using a three step procedure. Non-woven PET fabrics (DuPont, Spain) were cut into a size of 3 cm × 3 cm before the silver immobilization process. At first, an organosilicon thin film was deposited on the surface of the fabrics using the plasma deposition system. This 70 nm layer is used as a reservation layer for the silver immobilization and to control the silver nanoparticles adhesion to the PET fibres. The thickness of the reservation and barrier layers in this work was determined in the set of independent experiments on silicon wafer as it was described in our previous work.^36^

In the following steps, the samples with the plasma-polymerized layer on top were immersed into a suspension of AgNPs in ethanol and raised for drying. Silver nanoparticles (SSNANO, USA) of 20 nm size with a purity of 99.95% (trace metal basis) are used throughout the experiments as purchased. The color of the fabric changed from white to gray after the incorporation of the AgNPs. In the final step of the process, a second layer of organosilicon film was deposited using the plasma jet system. This second layer is used as a barrier to prevent the release of AgNPs. Two different thicknesses of the barrier layer film (10 and 50 nm) will be tested in the present study.

### Surface Characterization of silver loaded PET fabrics

The surface chemistry of the PET fabrics is determined by X-ray photoelectron spectroscopy (XPS) on a Versaprobe II system (Physical Electronics (PHI), USA) equipped with a monochromatic Al K_α_ X-ray source (hν = 1486.6 eV) operating at 23.3 W. The pressure in the analyzing chamber is maintained below 10^−6^ Pa during analysis and the photoelectrons are detected with a hemispherical analyzer positioned at an angle of 45° with respect to the normal of the sample surface. Survey scans and individual high resolution scans (C1s, O1s and Si2p) are recorded with a pass energy of 117.4 eV and 23.5 eV respectively. Elements present on the PET surfaces are identified from XPS survey scans, which have been performed on 3 different analyzing areas (200 *μ*m x 600 *μ*m) per sample. The obtained elements are quantified with Multipak software using a Shirley background and applying the relative sensitivity factors supplied by the manufacturer of the XPS instrument. Multipak software is also applied to curve fit the high resolution C1s peaks after the hydrocarbon component of the C1s spectrum (285.0 eV) is used to calibrate the energy scale. In a next step, the peaks were deconvoluted using Gaussian-Lorentzian peak shapes and the full-width at half maximum (FWHM) of each line shape was constrained between 1.3 and 1.8 eV.

SEM analysis is performed in order to investigate the morphology of the AgNPs in the PET samples. The samples are Au coated with a JFC-1300 Auto Fine Coater (JEOL, Belgium) in order to avoid charge effects. After applying the gold coating, the PET fabrics are studied with an InTouch Scope JSM-6010 SEM device (JEOL, Belgium).

### Antimicrobial efficiency of the silver loaded PET fabrics

Before the assay, all samples were sterilized by a 30 minutes UV exposure. The microorganisms were grown on Tryptic Soy Agar (TSA) (Oxoid, Drongen, Belgium) (*P. aeruginosa* and *S. aureus*) or Sabouraud agar (Sab) (BD, Franklin Lakes, NJ) (*C. albicans*) under aerobic conditions at 37 °C. Using a sterile forceps, the samples were placed in the wells of a 24-well microtiter plate and subsequently 1 ml of the cell suspension, containing approx. 10^4^ colony forming units (CFU)/ml was added. The plates were incubated for 24 h at 37 °C. Following incubation, the samples were transferred to 10 ml 0.9% (w/v) NaCl and subjected to three cycles of 30 s vortex mixing and 30 s sonication. Tenfold serial dilutions were made in 0.9% (w/v) NaCl and the number of CFU was determined by plate counting. To this end, one ml of each dilution was plated on TSA or Sab and the plates were incubated at 37 °C for 48 h.

## Author Contributions

X.D, and A. N. designed and performed most the experiments, and analyzed the data, figures and wrote the manuscripts. T. C. involved in the microbiology tests and analyses. P.C. and G. Z. provide the XPS and SEM measurements and analyses, respectively. R. M., N. D. and C. L. discussed the results, reviewed and commented on the manuscripts.

## Additional Information

**How to cite this article**: Deng, X. *et al*. Antimicrobial nano-silver non-woven polyethylene terephthalate fabric via an atmospheric pressure plasma deposition process. *Sci. Rep.* **5**, 10138; doi: 10.1038/srep10138 (2015).

## Supplementary Material

Supporting Information

## Figures and Tables

**Figure 1:**
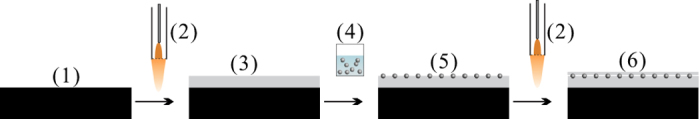
Scheme of the fabrication process. (1)original sample of non-woven PET fabric; (2)plasma jet system for thin film deposition, detail of the experimental setup is given as a supplement information; (3) 1^st^ step: plasma deposition of the reservation layer for silver nanoparticles immobilization and to control the silver nanoparticles adhesion to the PET fibres.; (4) prepared AgNPs dispersion; (5) 2^nd^ step: AgNPs adhesion on the surface of the samples by immersing into the dispersion; (6) 3^rd^ step: plasma deposition of the barrier layer.

**Figure 2:**
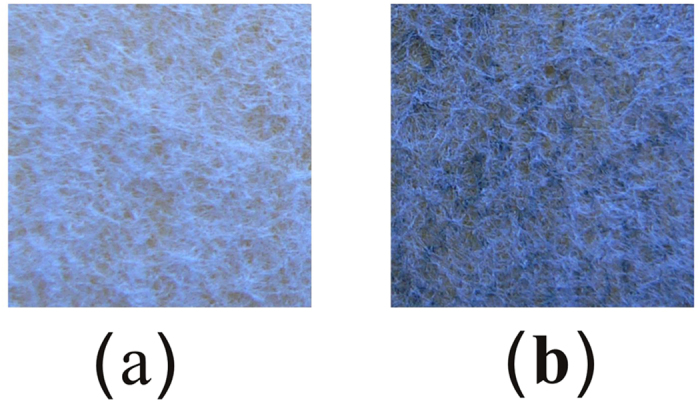
Visual change of the PET non-woven fabrics. (**a**) The PET non-woven fabrics before treatment; (**b**) the samples with AgNPs composite coating.

**Table 1:** Atomic composition of raw fabric, top side and bottom side of treated samples.

**Samples**	**C [at.%]**	**O [at.%]**	**Si [at.%]**
Raw fabric	73.4 ± 0.7	26.6 ± 0.8	0
Top side of treated sample	24.4 ± 0.8	54.0 ± 0.7	21.6 ± 0.6
Bottom side of treated sample	25.1 ± 1.1	53.4 ± 0.9	21.4 ± 0.7

**Table 2:** Atomic composition of the samples at different experimental steps and with variation of barrier layer thickness.

**Samples**	**C [at.%]**	**O [at.%]**	**Si [at.%]**	**Ag [at.%]**
Pure fabric	73.4 ± 0.7	26.6 ± 0.8	0	0
With 1st layer	24.4 ± 0.8	54.0 ± 0.7	21.6 ± 0.6	0
With incorporation of AgNPs	37.9 ± 0.5	45.2 ± 0.7	15.2 ± 0.8	1.9 ± 0.3
With 10 nm 2nd layer	25.3 ± 0.6	53.7 ± 1.0	19.9 ± 0.8	1.1 ± 0.2
With 50 nm 2nd layer	24.7 ± 0.4	53.8 ± 0.8	21.1 ± 1.0	0.5 ± 0.1
