# Supplementary material for: Antimicrobial nano-silver non-woven polyethylene terephthalate fabric via an atmospheric pressure plasma deposition process
Source: Sci Rep. 2015 May 7;5:10138. doi: 10.1038/srep10138 (PMC4423426; doi:10.1038/srep10138)
Supplement: Supporting Information [file srep10138-s1.pdf]

Supplementary information

Antimicrobial nano-silver non-woven polyethylene terephthalate fabric via an atmospheric pressure plasma deposition process

Xiaolong Deng[1], Anton Yu Nikiforov[1], Tom Coenye[2], Pieter Cools[1], Gaelle Aziz[1], Rino Morent[1], Nathalie De Geyter[1] and Christophe Leys[1]*

[1]Department of Applied Physics, Ghent University, Sint-Pietersnieuwstraat 41B4, 9000 Gent, Belgium, [2]Department of Pharmaceutical Analysis, Ghent University, 9000 Gent, Belgium

*Corresponding author Xiaolong.deng@ugent.be

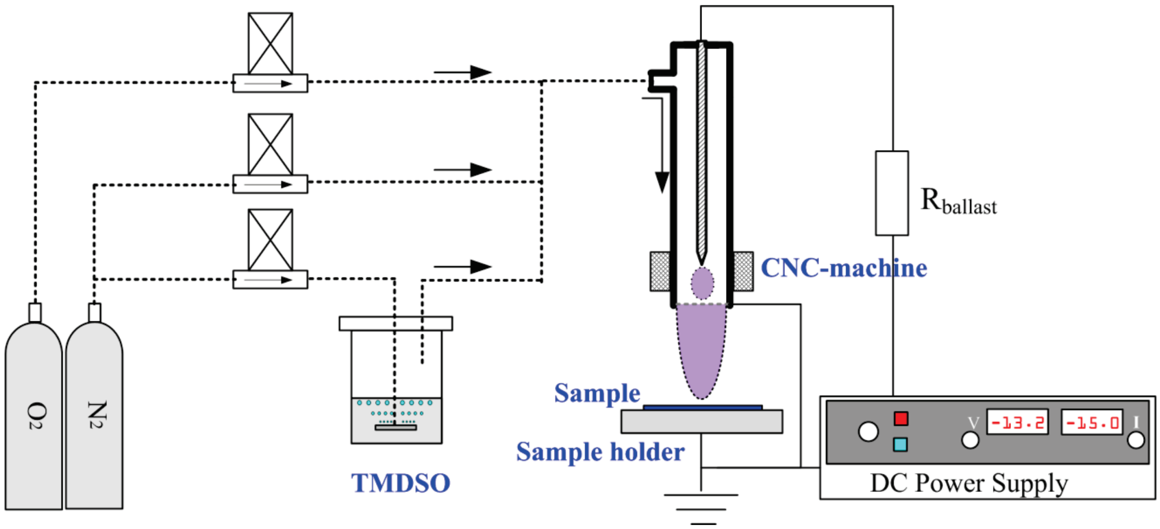

Figure S1. Experimental setup of the plasma deposition system. The non-woven fabric was put on a sample holder. The plasma head was mounted on a robotic arm which controlled by code program to scan the surface and to have a large scale uniform treatment.
